# Gene Therapy Using Nanocarriers for Pancreatic Ductal Adenocarcinoma: Applications and Challenges in Cancer Therapeutics

**DOI:** 10.3390/pharmaceutics14010137

**Published:** 2022-01-06

**Authors:** Eun-Jeong Won, Hyeji Park, Tae-Jong Yoon, Young-Seok Cho

**Affiliations:** 1 Laboratory of NanoPharmacy, College of Pharmacy, Research Institute of Pharmaceutical Science and Technology (RIPST), Ajou University, 206 Worldcup-ro, Yeongtong-gu, Suwon 16499, Korea; won4456@naver.com (E.-J.W.); tjyoon@ajou.ac.kr (T.-J.Y.); 2 Division of Gastroenterology, Department of Internal Medicine, Seoul St. Mary’s Hospital, College of Medicine, The Catholic University of Korea, Seoul 06591, Korea; phj8637@naver.com

**Keywords:** pancreatic ductal adenocarcinoma, miRNA, siRNA, CRISPR/Cas9, nanocarrier

## Abstract

Pancreatic ductal adenocarcinoma (PDAC) is one of the most lethal cancers worldwide, and its incidence is increasing. PDAC often shows resistance to several therapeutic modalities and a higher recurrence rate after surgical treatment in the early localized stage. Combination chemotherapy in advanced pancreatic cancer has minimal impact on overall survival. RNA interference (RNAi) is a promising tool for regulating target genes to achieve sequence-specific gene silencing. Here, we summarize RNAi-based therapeutics using nanomedicine-based delivery systems that are currently being tested in clinical trials and are being developed for the treatment of PDAC. Clustered regularly interspaced short palindromic repeats (CRISPR)/CRISPR-associated protein 9 (Cas9) genome editing has been widely used for the development of cancer models as a genetic screening tool for the identification and validation of therapeutic targets, as well as for potential cancer therapeutics. This review discusses current advances in CRISPR/Cas9 technology and its application to PDAC research. Continued progress in understanding the PDAC tumor microenvironment and nanomedicine-based gene therapy will improve the clinical outcomes of patients with PDAC.

## 1. Introduction

Pancreatic ductal adenocarcinoma (PDAC) is the third leading cause of cancer related deaths in the Unites States and the seventh leading cause worldwide. Its incidence is increasing by 0.5% to 1.0% per year, and it is thought it will be the second leading cause of cancer-related death by 2030 [1]. As its early diagnosis is very difficult, approximately 80% of patients present with locally advanced or metastatic disease [2]. PDAC often shows resistance to several therapeutic modalities and a higher recurrence rate after surgical treatment [3]. Adjuvant chemotherapy with 5-fluorouracil, leucovorin, irinotecan, and oxaliplatin (FOLFIRINOX), without bolus fluorouracil (modified FOLFIRINOX) leads to a median overall survival (OS) of 54.4 months among patients with resected pancreatic cancer [4]. However, combination chemotherapy such as FOLFIRINOX or nab-paclitaxel plus gemcitabine in advanced pancreatic cancer has minimal impact on OS in the range of weeks to months [5,6]. In addition, a number of clinical trials have tested pathway-specific targeted therapeutic agents, such as vascular endothelial growth factor inhibitors and multi-kinase inhibitors, alone or combined with conventional chemotherapy in metastatic pancreatic cancer but failed to demonstrate clinical meaningful benefits [7]. Although immune checkpoint inhibitors, such as cytotoxic T lymphocyte protein 4 (CTLA4) and programmed cell death protein 1 (PD-1) have shown promise for the treatment of multiple cancers, these agents demonstrate limited responses for the treatment of patients with PDAC, probably due to the multiple immune-regulatory pathways within the pancreatic tumor microenvironment (TME) [8].

At present, the initiating mechanisms of PDAC are relatively well understood. Most PDACs develop from premalignant lesions called pancreatic intraepithelial neoplasias (PanINs), which progress stepwise from low grade to high grade in types 1, 2, and 3. Subsequently, they progress to invasive lesions with the accumulation of various genetic alterations. Approximately 90% of PanINs of all grades have point mutations in the *KRAS* oncogene (particularly within codon 12). In addition, mutational inactivation of tumor suppressor genes, including cyclin-dependent kinase inhibitor 2A, tumor protein 53 (*TP53*), and SMAD family member 4 (*SMAD4*), are frequently detected in type 2 and 3 lesions. These findings suggest that *KRAS* mutations are associated with tumor initiation, and subsequent gene mutations are a rate-limiting step for tumor progression [2,9]. However, the molecular mechanisms underlying metastatic spread still need to be clarified. The TME of PDAC is heterogeneous and characterized by dense stroma, which consists of proliferating myofibroblasts (pancreatic stellate cells), extracellular matrix proteins and tumor vasculature, and inflammatory cells, including macrophages, mast cells, plasma cells, and lymphocytes [10]. Extensive desmoplastic stroma and severe hypovascularity of PDAC make it difficult to effectively deliver therapeutic agents to PDAC cells and are associated with poor prognosis and increased tumor invasion and metastatic spread [11]. A number of therapeutic agents to target or modify the TME are currently being evaluated but have shown poor and contradictory results due to the multi-faceted nature of tumor stroma. Genomic profiling has made novel therapeutics feasible for a small subset of patients, but current approaches require further testing in larger clinical trials [12].

Nanotechnology has led to significant advances in the diagnosis and treatment of various malignancies. To successfully treat PDAC, it requires effective delivery of drugs into the TME as well as tumor cells. Delivery using nanomaterial-based carrier systems enhances the antitumor activity of conventional chemotherapeutic agents including gemcitabine, fluorouracil, doxorubicin, and paclitaxel. The development and application of these drugs for the treatment of pancreatic cancer have been comprehensively reviewed elsewhere [13]. In addition, there have been great advances in the development of efficient delivery systems for gene therapy for the cancer treatment. Non-viral gene delivery systems are highly effective and are safer and easier to synthesize than delivery systems using viral vectors. For successful gene therapy, a number of delivery systems including liposomes, polymers, and inorganic nanoparticles have been developed and investigated with cancer-targeting moieties [14].

In this review, we present RNA interference (RNAi)-based therapeutics using nanomedicine-based delivery systems that are currently being investigated in clinical trials for the treatment of PDAC. Recently, we developed a novel therapeutic strategy that targets *KRAS* and *TP53* mutations at same time via liposome delivery of clustered regularly interspaced short palindromic repeats (CRISPR)/CRISPR-associated protein 9 (Cas9), ribonucleoprotein (RNP) complexes, and single-guide RNA (sgRNA) to overcome drug-resistance of PDAC, and this treatment significantly enhanced the anti-tumor activity of gemcitabine [15]. In addition, current advances in the application of genome editing technology to PDAC research are reviewed. Finally, we provide our perspective on the development of gene therapy using nanotechnology for future clinical translation.

## 2. RNAi Therapy for Pancreatic Cancer

RNAi is a process that regulates target genes using double-stranded RNAs (dsRNAs), which bind to sequences complementary to a gene’s coding sequence, resulting in the degradation of corresponding mRNAs and subsequent inhibition of translation to proteins. It can be achieved using three major different types of RNAi molecules: small interfering RNA (siRNA), microRNA (miRNA), and short hairpin RNA (shRNA). RNAi is initiated by Dicer enzyme mediated processing of dsRNA to smaller fragments (~22 nucleotides) of siRNA, which are then incorporated into RNA-induced silencing complexes [16]. Gene therapy using RNAi therapeutics is promising and improves precise gene delivery to treat human diseases including various cancers. Patisiran is an siRNA enveloped in lipid NPs, which inhibits the hepatic synthesis of transthyretin (TTR) and improves several clinical manifestations of hereditary TTR-mediated amyloidosis (HTA) [17]. It was approved in 2018 as the first RNAi-based therapy by the United States Food and Drug Administration and European Union for the treatment of HTA in adults [18]. However, there are several limitations of RNAi-based therapies, including easy nuclease-induce degradation within body fluids such as serum and inefficient delivery to desired cells, tissues, and organs. To overcome these problems, various nanocarriers have been developed, and these are highly attractive since they are safer and easier to synthesize than delivery systems using viral vectors. They fall into two categories: organic complexes (lipid complexes, conjugated polymers, and cationic polymers) and inorganic NPs (magnetic NPs, quantum dots, carbon nanotubes, and gold NPs).

### 2.1. RNAi Using miRNAs

miRNAs are 18–25 nucleotides in length and are endogenous non-coding RNA molecules that bind, at least partially, to complementary mRNA sequences, subsequently inducing target mRNA cleavage and degradation. Unlike siRNA, miRNAs can regulate multiple mRNAs rather than just one. miRNAs play significant roles in the expression of genes involved in cancer initiation, growth, progression, metastasis, drug resistance, and therapeutic efficacy. In addition, those secreted by exosomes from cancer cells regulate intercellular communication processes in the TME [19]. In PDAC, some miRNAs aberrantly express and regulate cancer initiation, progression, and invasion.

miR-21 functions as an oncogene and plays significant roles in cancer cell proliferation, differentiation, and survival in addition to cancer initiation and progression, which suggests that it may be a promising therapeutic target for PDAC [20]. miR-21, miR-196a, miR-196b, and let-7i are highly expressed and strongly associated with low survival in patients with PDAC [21,22]. Researchers have developed anti-miR-21 oligonucleotide-loaded tumor-penetrating complexes that consist of a C-terminal cell-penetrating peptide iRGD and an N-terminal fatty acid group to enhance hydrophobic interactions for the self-assembly of NPs (TPN-21). TPN-21 inhibits miR-21 expression and PDAC growth in PDO and upregulates phosphatase and tensin homolog and programmed cell death 4, resulting in strong suppression of tumor growth. Li et al. developed polyethylene glycol-polyethylenimine-magnetic iron oxide NPs conjugated with anti-CD44v6 single chain variable fragments for co-delivery of miR-21 antisense oligonucleotides and gemcitabine into PDAC cells [23]. These NPs induce apoptosis and inhibit tumor growth and metastasis in vitro and in vivo, suggesting synergistic anti-tumor effects of miR-21 gene silencing and chemotherapy in PDAC.

Xie et al. developed a local delivery system combining the silencing of miR-210, *KRAS^G12D^*, and blockade of C-X-C motif chemokine receptor 4 (CXCR4) with cholesterol- modified polymeric CXCR4 antagonist (PCX) NPs for the co-delivery of anti-miR-210 and siKRAS^G12D^ [24]. Cholesterol is conjugated to improve the efficacy of enhanced permeability and retention (EPR)-independent delivery. PCX NPs block cancer-stroma interactions, anti-miR-210 inactivates stroma-producing pancreatic stellate cells, and siKRAS^G12D^ kills pancreatic cancer cells. In this study, NPs were delivered via intraperitoneal (IP) administration to an orthotopic PDAC mouse model as an effective EPR-independent approach for targeting peritoneal tumors. There was nearly 15-fold higher tumor accumulation of NPs with local IP delivery compared to intravenous (IV) delivery.

Wu et al. revealed that miR-9 is positively associated with doxorubicin (DOX) sensitivity and developed supramolecular NPs condensed with an arginine-based plectin-1 (PL-1)-targeting chimeric peptide to bind RNA and target PDAC. PL-1/miR-9 NPs significantly enhanced DOX efficacy in PDAC cells and tumor growth from PDXs, which suggests that miR-9/eIF5A2 might be a novel potential drug for the synergistic therapy of PDAC [25].

miRNA-150 is a tumor suppressor that is downregulated in the majority of human pancreatic cancer tissues, which suggests that restoration of miR-150 might be a therapeutic strategy for pancreatic cancer [26]. Arora et al. developed poly (D,L-lactide-co-glycolide) (PLGA)-based nanoformulation (NF) for the delivery of miR-150 to pancreatic cancer cells, and treatment with it resulted in growth, clonogenicity, motility, and invasion via significant downregulation of target gene mucin 4 in vitro [27].

Although several preclinical studies have demonstrated the efficacy of miRNA-based gene therapy for the treatment of PDAC, many attempts at developing miRNA therapeutics have not been successful in clinical trials [28]. In addition, clinical trials for miRNA-based therapeutics have not been performed for the treatment of PDAC to date.

### 2.2. RNAi Using siRNAs

siRNA is a short non-coding ds-RNA of 21–23 nucleotides, which can induce RNAi silencing without eliciting non-specific interferon after introduction into the mammalian cells. It has the potential to silence genes encoding proteins that cannot be controlled by small molecules and programmable drugs [29]. Non-viral delivery systems using NPs enhance accumulation in tumor cells via EPR resulting from several factors including escape from the immune system, improved target to tumor and cell uptake, and prolonged circulation time [30]. Over the past two decades, a number of NP-based siRNA therapeutics have been developed and are being investigated in clinical trials [31]. In this section, recent advances in RNAi therapeutics using siRNA are described, focusing on siRNAs currently in clinical trials (Table 1).

Approximately 90% of PDAC patients have a KRAS mutation, which plays a major role of PDAC initiation. Kamerkar et al. developed exosomes derived from normal fibroblast mesenchymal cells carrying siRNA or shRNA for silencing KRASG12D [37]. IP injection of these engineered exosomes inhibited tumor growth and increased survival compared to liposomes due to their CD47-mediated protection from phagocytosis by macrophages and monocytes. A Phase I trial of patients with metastatic pancreatic cancer with KRASG12D mutation is underway (NCT03608631). Glutathione-S-transferase (GSTP) is a phase II detoxifying enzyme related to cell integrity maintenance, oxidative stress, and protection against DNA damage. It is a regulator of proteins involved in RAS signaling pathways and is highly expressed in several cancers with KRAS mutation including lung, colorectal, and pancreatic cancers [39]. NBF-006, a lyophilized lipid NP, consists of an ionizable, non-immunogenic, biodegradable lipid enveloping GSTP siRNA. Treatment with NBF-006 significantly inhibits tumor growth in KRAS mutant non-small cell lung cancer (NSCLC) xenograft models and prolongs survival in surgically implanted orthotopic lung tumor mice without toxicity [38]. It has recently entered a Phase I trial for previously treated NSCLC with KRAS mutation and previously treated progressive or metastatic NSCLC, pancreatic, and colorectal cancer (NCT03819387).

Khvalevsky et al. developed the first local prolonged siRNA delivery system (called the Local Drug EluteR, or LODER), which is a miniature biodegradable polymeric matrix that releases siRNA over a period of few months after administration to the tumors [40]. Treatment with LODER-encapsulated anti-KRASG12D siRNA (siG12D LODER) inhibits cancer cell proliferation and epithelial-mesenchymal transition with significant decrease in KRAS levels and inhibits orthotopic pancreatic tumor growth and prolonged mouse survival in vivo. In a Phase 1/2a clinical trial that included 15 patients with inoperable PDAC, treatment with standard of care chemotherapy following siG12D LODER insertion into the tumor with a needle during an endoscopic ultrasound biopsy procedure resulted in stable disease in 10 patients and a partial response in 2 patients with favorable safety data (NCT01188785) [35]. In an ongoing trial that started in 2017, 80 patients with unresectable or borderline resectable locally advanced pancreatic cancer have been assigned to receive repeated doses of 2.8 mg siG12D LODER with chemotherapy (gemcitabine and nab paclitaxel or FOLFIRINOX) or chemotherapy alone (NCT01676259).

Ribonuclease reductase M2 subunit (RPM2) is correlated with biological behaviors of tumor cells including proliferation, invasion, migration, cell cycle, and apoptosis and plays an important role in tumorigenesis in several cancer types [41]. CALLA-01, a targeted NP system, consists of cyclodextrin-containing polymer, polyethylene glycol (PEG), human transferrin as a targeting ligand for binding transferrin receptors, and siRNA designed to reduce expression of RPM2 for tumor inhibition and/or tumor size reduction. In 2008, it entered the first human Phase I trial involving systemic administration of siRNA to patients with solid cancer including PDAC (NCT00689065). These NPs were successfully delivered into cancer cells, and it was confirmed by cancer cells containing nanoparticles in tumor biopsies from patients with melanoma after systemic administration. In addition, the expression of RPM2 mRNA and protein is reduced in patients who receive the highest doses, which suggests that specific gene inhibition by an RNAi might be one cancer treatment modality [42]. In a Phase Ia/Ib trial, CALLA-01 showed similar safety profile in 24 patients with different cancers compared to animals [32].

Atu027 is an RNAi therapeutic formulation based on cationic lipids. It contains neutral fusogenic, PEG-modified lipid components and siRNA molecules that specifically target protein kinase N3 in the vascular endothelium, a downstream effector of the phosphoinositol-3-kinase signaling pathway [43]. Atu027 inhibits tumor growth and lymph node metastasis in orthotopic mouse models for prostate and pancreatic cancer mouse models and hematogenous metastasis in mouse models for spontaneous lung cancer [43,44]. In the first human Phase I clinical trial of this compound (NCT01808638), 10 escalating doses of Atu027, as a single IV administration, followed by twice weekly doses for a 28-day cycle, were given to 24 patients with advanced solid tumors. The treatment was safe and resulted in tumor stabilization in 41% of patients. In addition, most patients had a reduced soluble variant of vascular endothelial growth factor receptor-1, which suggests its potential as a biomarker [33]. In a Phase II trial, 23 patients with metastatic pancreatic cancer received combination treatment of gemcitabine and Atu027 (NCT01808638). The treatment was safe, and twice weekly Atu027 led to significantly improved progression-free survival [34].

Polo-like kinase 1 (PLK1), an essential cell-cycle protein, plays multiple roles in mitosis and cytokinesis. It is overexpressed in various types of cancer and negatively affects patient outcome. In addition, inhibition of PLK1 expression induces mitotic arrest and apoptosis, resulting in tumor growth inhibition [45]. Stable nucleic acid lipid particles (SNALPs) are effective siRNA delivery system, and do not need active targeting moieties due to passive disease-site targeting. In a preclinical liver tumor model, SNALP-formulated PLK1 siRNA (TKM-080301) demonstrated potent antitumor activity and induced no measurable immune reaction, minimizing potential nonspecific effects [36]. A Phase I clinical trial of TKM-080301 was performed in patients with hepatic metastasis from colorectal, pancreas, gastric, breast, and ovarian cancer, but it was terminated without report of results (NCT01437007).

The unique TME of the PDAC make it difficult to deliver therapeutic agents to the tumor cells and raises the need for the development of a novel therapy. Although the role of hypoxia-inducible factor 1 (HIF1, including HIF1α and HIF1β) in PDAC development mechanisms is not completely understood, HIF1α stabilization in the hypoxic TME is associated with transcriptional activation of multiple signaling pathways involved in the regulation of cell survival, tumor invasion, angiogenesis, and metabolism [46]. Zhao et al. developed lipid-polymer hybrid NPs (LENPs), which consist of a single layer or bilayer lipid shell around a polymeric core made from cationic ε-polylysine co-polymer (ENPs) [47]. Negatively charged si-HIF1α is absorbed on the surface of ENPs and gemcitabine encapsulated to the hydrophilic core. LENPs protected NP aggregation, have prolonged lifetimes with a half-lifetime longer than 3 h, and improved drug release through an enhanced tumor vasculature effect within tumor tissues. Treatment with LENP-Gem-si-HIF1α results in effective silencing of HIF1α both in vitro and in vivo as well as significant synergistic tumor growth inhibition. Another hypoxia-associated major transcription factor, HIF2α (also called endothelial PAS domain protein 1 [EPAS1]) in pancreatic cancer, was targeted using NPs with siRNA loaded onto a polyethylenimine-poly(lactide-coglycolide) (PLGA)/poloxamer [48]. Treatment inhibited PDAC cell proliferation by apoptosis induction under hypoxic conditions in vitro, and significantly inhibited microvascular formation and tumor growth in orthotopic PDAC mice.

The cytoskeleton, which included microtubules, is composed of α- and β-tubulin heterodimers. Increased βIII-tubulin expression is associated with poor OS and drug resistance in various types of cancer including pancreatic cancer, and silencing of βIII-tubulin with shRNA leads to tumor growth inhibition and enhanced sensitivity to chemotherapy in vitro and in vivo, which suggests the possibility of a novel therapeutic target for PDAC [49]. The limitations of using highly charged cationic NPs as a delivery system for siRNA include their toxicity and potential to interact with serum proteins in the bloodstream. To overcome this problem, Teo et al. developed star polymers with different lengths of cationic poly(dimethylaminoethyl methacrylate) sidearms and varied amounts of poly(oligo(ethylene glycol) methyl ether methacrylate), which were highly accumulated in orthotopic PDAC tumors in mice and had a silencing efficiency of 80% at the gene and protein levels [50].

## 3. CRISPR-Cas Gene Editing 

CRISPR functions as an immune system in *E. coli* [51,52]. Recently, the CRISPR systems have been developed as tools in gene therapy and have shown good target gene specificity, high editing efficiency, and research simplicity compared to zinc finger nuclease and transcription activator-like effector nuclease [53,54]. CRISPR/Cas9 is an RNA-guided endonuclease consisting of Cas9 protein and sgRNA with target sequence specificity for DNA cleavage. DNA damage induced by Cas9 is repaired through a non-homologous end-joining DNA repair pathway or homology-directed repair pathway (HDR), which are cellular DNA repair mechanisms [55,56]. CRISPR variants have been developed based on this system. Base editing system (cysteine and adenine base editor) is composed of a single-strand DNA nickase and deaminase, which can be applied for the treatment of diseases attributable to a point mutation [57,58,59]. Cas13 (previously referred to as C2C2) edits RNA and reduces the risk for DNA damage due to off-target effects [60,61]. The process of prime editing involves DNA nickase and a reverse transcriptase enzyme, which generates new DNA by duplicating an external RNA template [57,62]. It can lead to the formation of indels (insertions, deletions) and base conversions without the DNA double-strand breaks or donor DNA templates. However, it needs an appropriate delivery system for the treatment of cancer. Viral vectors (retrovirus, adenovirus, adeno-associated virus vector) are popular in the gene therapy field [63,64,65] but their clinical use is limited by several factors, such as the increased adaptive immune response from neutralizing antibodies by the capsids of adeno-associated virus, the potential immunogenicity, and gene mutations by their chromosomal insertion [66,67,68,69,70]. Therefore, an ideal vehicle would show target specificity, minimize off-target efficiency, and retain non-immunogenicity.

**Figure 1:**
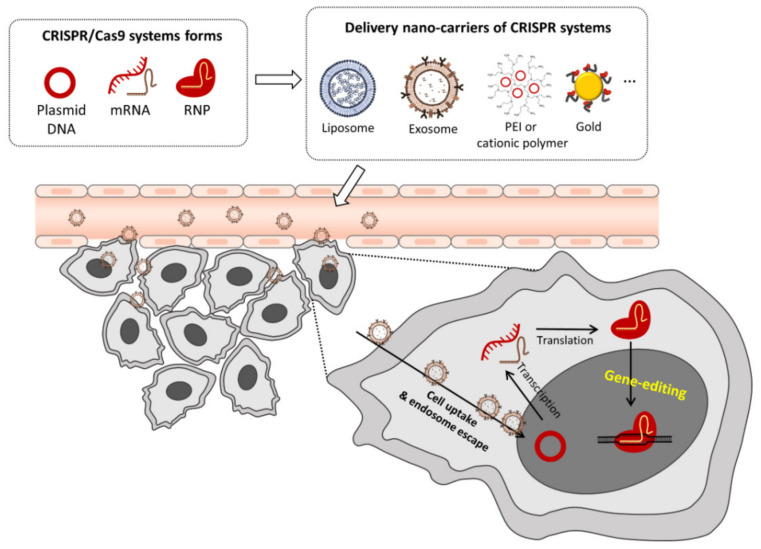
The cell delivery pathway of CRISPR/Cas9 encapsulated-nanoparticles. The CRISPR systems can be applied for gene therapy with non-viral carriers in three forms that include: plasmid DNA (Cas9/sgRNA plasmid), in vitro transcribed Cas9 mRNA/sgRNA, and pre-assembled ribonucleoprotein (RNP) complex. Such nanoparticles can take several forms, including PEGylated- or cationic lipid-NPs (liposome), cell-mediated exosome, cationic polymers (polyethyleneimine, PEI; protamine, PA), and cationic lipid- or polymer-coated gold NPs. The plasmid DNA, mRNA and RNP of NPs may enter the cell via endocytosis and endosome escape, processing the RNP expression and gene editing.

CRISPR systems can be applied in PDAC gene therapy using three types of nanocarriers: plasmid DNA (Cas9/sgRNA plasmid), in vitro transcribed Cas9 mRNA/sgRNA, and pre-assembled ribonucleoprotein (RNP) complex (Figure 1). In general, the successful delivery of three forms requires cationic coatings to fully condense them [71]. Negatively charged plasmid DNAs, mRNAs, and RNPs are coated with cationic molecules to form complexes with structural stability. The complexes bind to cell membranes through electrostatic interactions and are taken into cells via endocytosis. Typically, cationic lipid-based polymers (polyethylenimine, PEI) and peptides (protamine) form positively charged particles that improve the delivery efficiency and stability in vivo. Cationic lipid-based NPs have additional properties such as tumor targeting, co-encapsulation of chemotherapeutic agents, and others [72,73,74,75]. PEI and protamine are also the most commonly used cationic molecules in vitro and in vivo. They principally consist of high-density amine groups that interact with negatively charged nucleic acids or RNPs. They enhance intracellular delivery efficiency and endosomal escape at a lower pH due to the proton sponge effect [76,77]. In addition, inorganic materials, such as gold (Au) NPs, are attractive for the delivery of RNPs and donor DNA into cells for HDR repair. Au NPs are easily modified with thiol-terminated single-stranded DNA and bind RNPs via non-specific electrostatic forces [78,79].

### 3.1. Plasmid DNA (Cas9/sgRNA Plasmid) Delivery

Although viral vectors such as adenoviruses and lentiviruses are efficient delivery systems for CRISPR DNA, their immunogenicities make them potential carcinogens and they have considerable molecular weights [80]. Furthermore, the CRISPR/Cas9 mRNA and protein delivery remains challenging due to issues related to stability, encapsulation efficiency, and their high cost of production. CRISPR/Cas9 plasmid delivery has been used due to the low cost, storage stability, and the potential of prolonged expression. However, plasmid delivery risks genomic integration, cell stress, and off-target effects due to long-term transgene expression [81,82].

**Figure 2:**
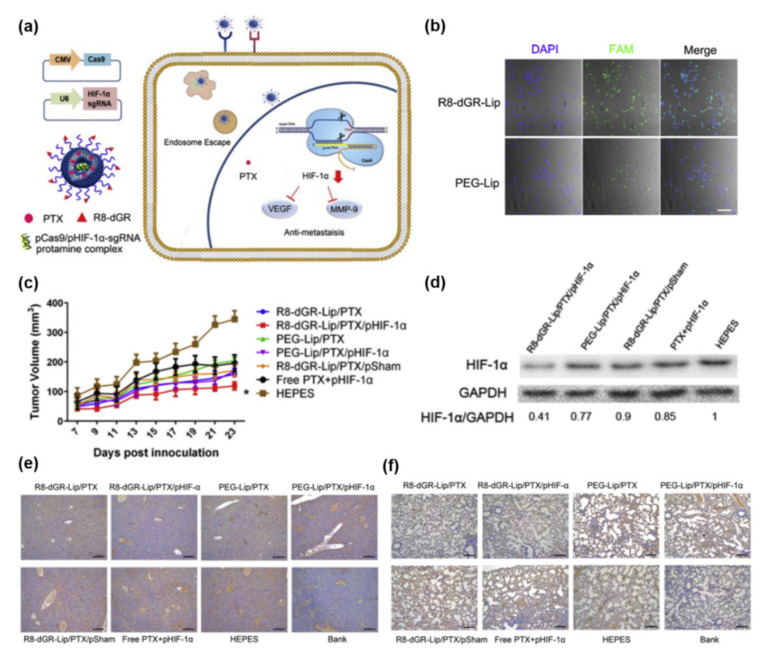
R8-dGR peptide modified and loaded CRISPR/Cas9-HIF-1α and paclitaxel cationic liposome for targeted pancreatic treatment. (**a**) Scheme of the structure of the R8-dGR-Lip/pHIF-1α and paclitaxel (PTX) delivery in pancreatic cancer cells. (**b**) Cellular internalization of with or without the R8-dGR peptide (Cys-RRRRRRRRdGR) on BxPC-3 cells. scale bar: 100 μm. (**c**) Tumor volume of BxPC-3 xenograft models treated with different formulations (mean ± SD, *n* = 10, * *p* < 0.05). (**d**) In vivo downregulation of HIF-1α by pHIF-1α in tumor tissues. (**e**,**f**) MMP9 expression in liver (**e**) and lung (**f**) of mice. Scale bar: 100 μm (adapted from Ref. [71]).

Plasmid DNA-encoded Cas9 nuclease and sgRNA-encapsulated lipid-based NPs have been successfully studied in vivo for PDAC treatment. Li et al. reported a tumor-targeted lipid-based CRISPR/Cas9 plasmid delivery system developed to suppress HIF-1α (Figure 2a) [71]. They synthesized the R8-dGR peptide (Cys-RRRRRRRRdGR) bound to integrin αvβ3 and neuropilin-1 in over-expressed tumor cells, and it enhanced in vivo targeting of pancreatic cancer. The peptide was utilized to lower the charge density of DOTAP-based cationic liposome. In addition, this cell penetrating peptide enhanced cell internalization and transfection efficiency (Figure 2b). HIF-1α over-expression in tumors is associated with aberrant p53 and upregulates angiogenesis and metastasis-related signals such as vascular endothelial growth factor (VEGF) and matrix metalloproteinase 9 (MMP9) [83,84]. Therefore, the Cas9-HIF-1α plasmid DNA/protamine complex and paclitaxel (PTX) co-encapsulated liposome to promote the anti-metastatic effects could suppress tumor growth in vivo (Figure 2c,d). Consequently, R8-dGR-Lip/pHIF-1α and PTX downregulated HIF-1α and its downstream molecules VEGF and MMP-9, leading to inhibition of metastasis to livers and lungs (Figure 2e,f).

**Figure 3:**
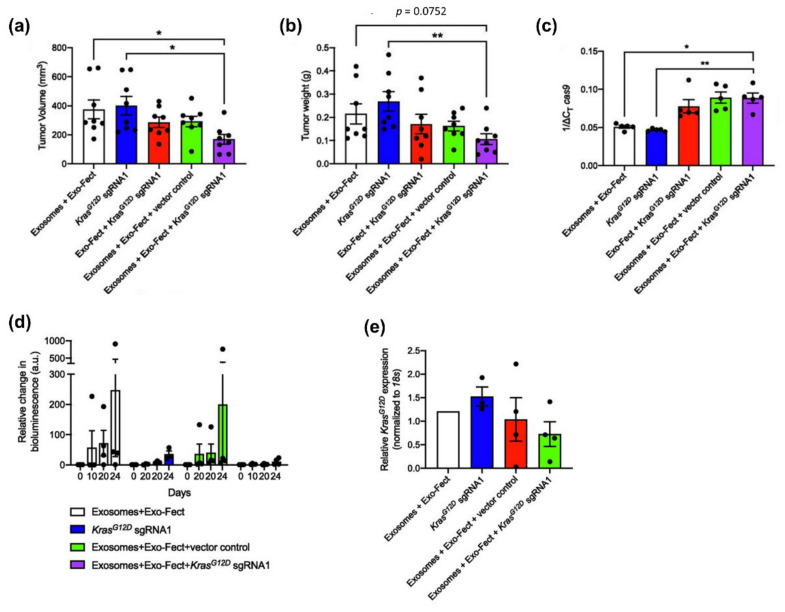
Exosome mediated treatment of CRISPR/Cas9 in as allograft and orthotropic model of pancreatic cancer. (**a**,**b**) Tumor volume (**a**) and tumor weight (**b**) after exosomes-Cas9/KRASG12D sgRNA intravenously administration in allograft models (KPC689 cells) every other day for 2 weeks (*n* = 8 mice in each group). (**c**) Cas9 mRNA expression levels in tumor tissues by quantitative PCR (normalized to 18S, *n* = 5 mice per group). (**d**) Tumor growth by bioluminescent imaging after exsomes-Cas9/KRASG12D sgRNA Intraperitoneal injection in orthotopic models (KPC689-GFP-Luciferase cells) every other day for 3 weeks. (**e**) KRASG12D mRNA expression levels at the end point in orthotropic tumor tissues (mean ± SD, * *p* < 0.05, ** *p* < 0.01) (adapted from Ref. [88]).

Exosomes are naturally released to nano-sized extracellular vesicles from all cells and contain DNA, RNA, metabolites, cell-surface protein, and lipids depending on the cell origin [85]. Unlike synthetic nanocarriers, exosomes originating from cells have the advantage of biocompatibility, non-immunogenicity, and non-cytotoxicity [86]. In addition, they are contained in various plasma membranes and have enhanced half-live [87]. Therefore, they are suitable as carriers of internal cargo to recipient cells. Recently, McAndrews et al. reported that exosomes could be successfully engineered to encapsulate CRISPR/Cas9 plasmid DNA and delivery it into tumor cells. The exosomes were derived from bone marrow-derived MSCs and did not show repeated dose toxicity [86]. They suppressed the mutant *KRAS^G12D^* oncogene, leading to inhibited tumor growth in a syngeneic allograft model and orthotropic model (Figure 3) [88].

### 3.2. In Vitro Transcribed Cas9/sgRNA mRNA Delivery

CRISPR/Cas9 mRNA delivery has advantages over plasmid DNA. The mRNA is not integrated into the target genome, which prevents off-target effects [65,89,90]. In addition, the mRNA leads to quick and transient Cas9 protein expression in the cytoplasm. However, mRNAs are large in size, unstable, and susceptible to degradation during delivery [91].

### 3.3. Pre-Assembled Ribonucleoprotein (RNP) Complex Delivery

CRISPR/Cas9 RNP does not need to be translated into cells. It is directly transported into the nucleus and edits the target gene. In addition, it has low toxicity, high gene-editing efficiency, low off-target effects, and no chance of integration into the genome of recipient cells [65,89,92,93]. The Cas9 protein and sgRNA can be simply mixed ex vivo for delivery to cells. The Cas9 protein/sgRNA complex is negatively charged. The complex can be positively charged by modification to facilitate cell membrane affinity and internalization [92,94,95,96,97]. In addition, bio-reducible disulfide bonds of nanocarriers can be adjusted to improve degradation and endosomal escape [98]. In addition, the Cas9 protein can be modified to enhance the encapsulation efficiency of NPs [99,100].

**Figure 4:**
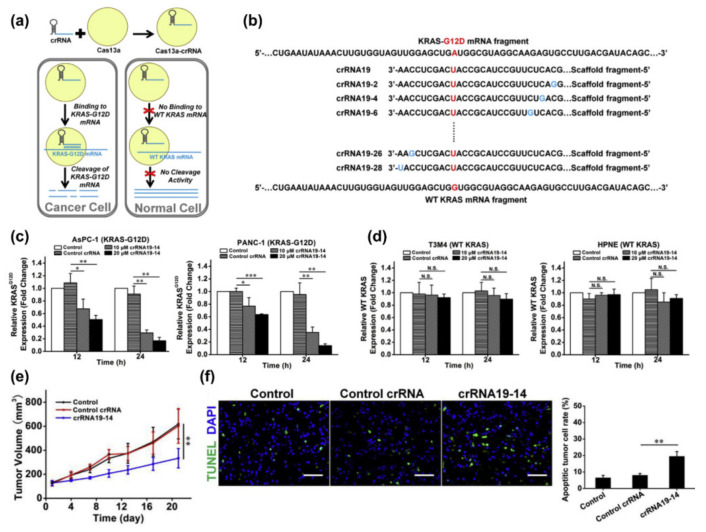
CRISPR/Cas13a-lipofectamine CRISPRMAX delivery for KRAS mutated pancreatic cancer treatment. (**a**) The scheme of Cas13 system differentiates wild type (WT, normal cells) and mutated KRAS genes (cancer cells). (**b**) crRNA sequences of the crRNA19-target sequence in KRAS-G12D and KRAS WT. (**c**) qRT-PCR analysis of KRAS-G12D mRNA expression after crRNA19-14 delivery into KRAS-G12D mutated cells. (**d**) qRT-PCR analysis of KRAS-WT mRNA expression after crRNA19-14 delivery into KRAS-WT cells. (**e**) The tumor volumes of subcutaneous AsPC-1 xenograft after administration with repeated intratumoral injections of the Cas13a-lipofectamine for 21 days. (**f**) Staining for apoptotic cells in tumor tissues (green fluorescence). Scale bar: 50 μm. (mean ± SD, * *p* < 0.05, ** *p* < 0.01, and *** *p* < 0.001) (adapted from Ref. [101]).

Zhao et al. studied CRISPR-Cas13a effector protein delivered to pancreatic cancer cells using lipofectamine CRISPRMAX and found that the Cas13a-crRNA complex bound to and cleaved target RNA and xenograft model by intratumoral injection (Figure 4a) [101]. CRISPR-Cas13a system reduces mRNA expression in mammalian cells and shows an improved efficacy and specificity over RNA interference [60,61]. The system reduced the KRAS^G12D^ mRNA expression with up to 70% knockdown efficiency (Figure 4b–d), leading to apoptosis and tumor growth inhibition (Figure 4e,f) [101].

**Figure 5:**
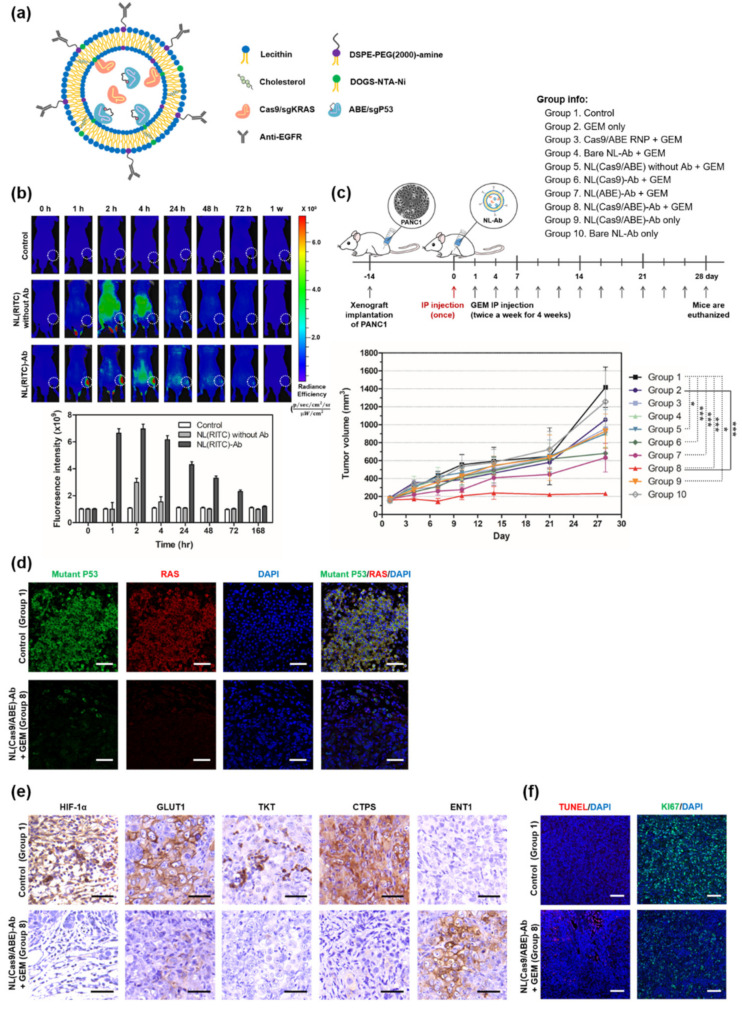
Dual-gene editing nanoliposome system as an overcoming drug-resistance for PDAC. (**a**) Graphical summary of NL(Cas9/ABE)-Ab composition. (**b**) Dynamic distributions of tumor targeting efficiency by NL with or without Ab in vivo. Mice were injected intraperitoneally once with NL-Ab labeled with RITC. PANC1 xenograft tumor was marked as white circles. (**c**) The scheme of PANC1 xenografts co-administrated with NL(Cas9/ABE)-Ab and gemcitabine. The NL(Cas9/ABE)-Ab was injected intraperitoneally once (red arrow) and then gemcitabine (50 mg/kg) was administered twice a week for 4 weeks. Mean ± SEM, *n* = 3 per group, * *p* < 0.05, *** *p* < 0.001. (**d**) Immunofluorescence with mutant P53 and KRAS on the tumor sections. Scale bar: 50 μm. (**e**) Immunostaining with HIF1a-related glucose metabolism and gemcitabine transporter (GLUT1, TKT, CTPS and ENT1) on the tumor. Scale bar: 50 μm. (**f**) Immunostaining with apoptotic cells (TUNEL assay and Ki67) by NL(Cas9/ABE)-Ab and gemcitabine on the tumor. Scale bar: 100 μm (adapted from Ref. [15]).

Recently, our group has developed a tumor targeted-nanoliposome (NL[Cas9/ABE]-Ab) that simultaneously encapsulates CRISPR/Cas9 and adenine-base editor (ABE) proteins for dual-gene editing (Figure 5) [15]. The Cas9 and ABE proteins are modified with his-tag protein to improve NL-encapsulation efficiency. In addition, we used PEG-bound lipid for structure stability and increased receptor-mediated endocytosis. Gemcitabine is used as first-line chemotherapy for PDAC but is limited in that most PDAC patients develop resistance to it [102]. The *KRAS* mutation and loss of P53 induces HIF-1α stabilization, which is the master regulator of glucose metabolism and a major driver of gemcitabine chemoresistance in PDAC [103,104]. Co-administration of NL(Cas9/ABE)-Ab and gemcitabine markedly inhibits tumor proliferation in a PANC1 xenograft (Figure 5c). NL(Cas9/ABE)-Ab inhibits *KRAS* and *TP53* mutations, and regulates HIF-1α-related glycolysis, which promotes gemcitabine sensitivity in vivo (Figure 5d–f). Therefore, the dual-gene editing tool of the *KRAS* mutation and mutant *TP53* might overcome drug-resistance in PDAC [15].

## 4. Conclusions and Future Perspectives

Prerequisites for successful gene therapy include rapid, simple, and easy synthesis, safety, and efficient delivery. Gene therapy using RNAi-based therapeutics and CRISPR genome editing is promising and can improve precise gene delivery to tumor tissue. Although there have been major advances in the development of gene therapy for the PDAC treatment, there is still much room for improvement in pharmacokinetics, pharmacodynamics, and methods to improve safety. RNAi-based therapeutics have several barriers (e.g., systemic circulation and targeted delivery must be improved, off-target effects must be minimized, etc.) and it is essential to further develop safe, biocompatible, and biodegradable NP-based therapeutics for clinical application. EPR of nanoparticle-based carriers make it easier to accumulate in the tumor cells. However, extracellular and intracellular barriers in PDAC are hurdles to overcome. Nanoparticles with appropriate particle size and their chemical modifications and functionalization with targeting ligands are necessary to enhance the uptakes into tumor cells and address other biological issues such as intracellular trafficking and intracellular siRNA escape [105]. In addition, combination treatment with chemotherapy and RNAi-based therapeutics or a strategy combining RNAi molecules and anticancer drugs would help overcome drug resistance and TME barriers.

CRISPR/Cas9 genome editing has widely been used not only in the development of cancer models and as a tool for the identification and validation of therapeutic targets but also as a potential cancer therapeutic [106]. The field of cancer immunotherapy is the most advanced clinical application. The first Phase I trial from China including 22 patients with NSLC used CRISPR-Cas9 PD-1-edited T cells from patient blood, and the treatments were safe and showed some therapeutic efficacy (NCT02793856) [107]. Several clinical trials are ongoing to demonstrate the efficacy of more potent chimeric antigen receptor T cells using CRISPR to knock out immune co-inhibitory pathways or signaling molecules [108]. However, there are several huddles to cross before CRISPR/Cas9 genome editing can be used in clinical practice, including potential off-target effects and safety issues. A novel therapeutic approach is required for efficient delivery, accurate targeting of desired cells, efficient gene editing, and to minimize off-target effects and immune responses. Although several clinical trials for gene therapy have not been successful, the progress in understanding of the PDAC TME and nanomedicine-based gene therapy can make it possible to improve the clinical outcomes of patients with PDAC.

## Figures and Tables

**Table 1:** Nanocarriers-based siRNA therapeutics in clinical trials for the treatment of pancreatic cancer.

Therapeutic siRNAs	Indication	Target Gene /Protein	Delivery Systems	Route of Administrations	Phase/Status	Reference
CALLA-01	Cancer, Solid tumors	RPM2	Cyclo-dextrin based polymer	Systemc/IV	I/Terminated	[32]
Atu027	Pancreatic cancer	PKN3	Liposomes (Lipoplexes, Cationic lipid)	Systemc/IV	II/Completed	[33,34]
siG12D LODER	Pancreatic cancer	KRAS^G12D^ mutation	Polymer matrix (LODER polymer)	Local/Surgical implantation	II/Recruiting	[35]
TKM 080301	Solid tumors with liver involvement	PLK-1	SNALP	Systemc/IV	II/Completed	[36]
iExosomes	Pancreatic cancer	KRAS^G12D^ mutation	Mesenchymal Stromal Cells-derived Exosomes	Systemc/IV	I/Recruiting	[37]
NBF 006	Non-small cell lung, Colorectal, and Pancreatic	GSTP	Lipid nanoparticles	Systemc/IV	I/Recruiting	[38]

Abbreviation: GSTP, Glutathione-S-transferase; IV, intravenous; PKN2, protein kinase N3; PLK1, polo-like kinase 1; RPM2, ribonuclease reductase M2; SNALP, Stable nucleic acid lipid particle.

## Data Availability

The data presented in this study are available in this manuscript.
